# Rich Pickings Near Large Communal Roosts Favor ‘Gang’ Foraging by Juvenile Common Ravens, *Corvus corax*

**DOI:** 10.1371/journal.pone.0004530

**Published:** 2009-02-25

**Authors:** Sasha R. X. Dall, Jonathan Wright

**Affiliations:** Centre for Ecology & Conservation, University of Exeter, Cornwall Campus, Penryn, Cornwall, United Kingdom; University of Utah, United States of America

## Abstract

Ravens (*Corvus corax*) feed primarily on rich but ephemeral carcasses of large animals, which are usually defended by territorial pairs of adults. Non-breeding juveniles forage socially and aggregate in communal winter roosts, and these appear to function as ‘information centers’ regarding the location of the rare food bonanzas: individuals search independently of one another and pool their effort by recruiting each other at roosts. However, at a large raven roost in Newborough on Anglesey, North Wales, some juveniles have been observed recently to forage in ‘gangs’ and to roost separately from other birds. Here we adapt a general model of juvenile common raven foraging behavior where, in addition to the typical co-operative foraging strategy, such gang foraging behavior could be evolutionarily stable near winter raven roosts. We refocus the model on the conditions under which this newly documented, yet theoretically anticipated, gang-based foraging has been observed. In the process, we show formally how the trade off between search efficiency and social opportunity can account for the existence of the alternative social foraging tactics that have been observed in this species. This work serves to highlight a number of fruitful avenues for future research, both from a theoretical and empirical perspective.

## Introduction

In the winter, common ravens (*Corvus corax*) typically forage over large areas on rich but ephemeral carcasses of large animals, which can be buried by unexpected snowfalls or consumed rapidly by other scavengers [1]. Originally, these would have been animals such as deer dying in the winter in mountains and forests. However, in modern day Europe they are often sheep in areas of extensive agricultural pasture [2]. Carcasses are usually discovered and defended by the local resident territorial pair of adult birds, and it normally requires groups of floating non-territorial juveniles to displace them [1], [3]–[6]. Non-breeding juveniles therefore tend to forage socially and aggregate in communal winter roosts, and there now appears to be mounting evidence that such roosts may act as ‘information centers’ regarding the location of food bonanzas [1], [4]–[10].

Ward and Zahavi [11] first suggested that communal roosts (and nests) have evolved to facilitate the exchange of foraging information. This information center hypothesis (ICH) has since stimulated a lot of empirical work, but it has also been criticized on logical grounds [12], [13]. To make sense of the conflicting theoretical arguments, and to separate out influences of the contrasting benefits of information sharing and group foraging, Dall [14] used evolutionary game theory to explore the problem. The formulation is based upon the North American raven system, studied by Heinrich and co-workers for many years [1], [4], [5], [7]–[10]. This system is thought to represent the original native winter habitat of the common raven, in which non-territorial juvenile birds forage for carcasses over large snow-covered forested areas and form transient communal overnight roosts. The large size of the animal carcasses involved and their temporary nature result in little net cost to foraging in groups as a result of competition for food. In the model, it is also assumed that pooling the independent search effort of individuals is the most effective way of locating rare food bonanzas, but groups that search together do better in gaining access to carcasses once they have been located. As in an earlier model by Mesterton-Gibbons and Dugatkin [15], Dall [14] confirms that the co-operative foraging behavior observed in juvenile common ravens – ‘search independently and recruit other individuals from the overnight roost’ – can be an evolutionarily stable strategy (ESS). Interestingly, the opportunity to share foraging information can be sufficient to drive this result, thereby confirming the logic of the ICH, while the benefits of foraging as a member of a group are not so necessary, but they are still likely to play an important role in the raven system.

In contrast to North America, raven roosts in Europe are far larger and more stable, probably as a result of the birds foraging on more abundant food and over much shorter distances in an agricultural landscape [2], [16]. At one of these very large raven roosts at Newborough on Anglesey in North Wales, Wright and co-workers [6] studied cooperative foraging strategies using sheep carcasses placed at varying distances from the roost that were baited with color-coded plastic beads. These beads were ingested at the carcass and regurgitated in aggregations of pellets back in the roost, the spatial distribution of which consistently reflected the geographical location of bait sites. This pattern was less distinct for nearby bait sites, probably because these sites were over flown by a greater number of birds fanning out from the roost to forage at more distant locations. In addition, aggregations of beads at the roost grew daily with an increasing radius centered upon the first pellet per carcass. This increase in pellet numbers mirrored the linear increase of ∼6 birds per day in the size of groups flying between roost and at the carcasses each morning. Interestingly, rates of recruitment were greater for carcasses closer to the roost suggesting that fewer birds were available and/or willing to be recruited to more distant baits [6]. Taken together, these results provide strong circumstantial evidence for large European raven roosts operating as structured information centers, confirming the results of Marzluff and co-workers [10] for the smaller more transient North American roosts.

Furthermore, as well as such data for the main Newborough roost, Wright et al. [6] present results from two ‘sub-roost’ groups of ∼30 birds each. These sub-roost groups roosted in separate locations close to the main roost. The birds within each sub-roost searched and foraged together as a coherent group, and there was no evidence for the additional recruitment to carcasses, as seen in the main roost birds. Wright et al. [6] suggest that, in highly competitive areas close to the main roost, group foraging may represent a strategic alternative to the usual individual searching and recruitment. Indeed, just such a ‘gang foraging’ strategy emerged as the only alternative ESS in the model by Dall [14]. However the conditions leading to its dominance over the typically observed recruitment-based foraging strategy have been explored only in passing since it was only presumed to be plausible ‘in theory’. The aim of this paper is to explore in detail the conditions favoring this newly observed yet theoretically anticipated juvenile common raven foraging behavior with particular reference to its existence in the Newborough raven roosts.

### The Model: Dall [14] revisited

Here we adapt the model of Dall [14] to elucidate why the conditions observed close to the Newborough roost [6] should favor ‘foraging in gangs’ instead of the search-individually-and-recruit foraging typical of juvenile common ravens [6], [7]. To this end, we make two assumptions in addition to those made in the original formulation. Firstly, we assume that the ‘search individually and recruit’ strategy is ancestral, and that the birds' behavioral responses evolved where roost membership was very transient and therefore birds were unlikely to find themselves at subsequent roosts with the same individuals or kin. This is reasonable since such conditions dominate the New England raven system [1], [4], [5], [7]–[10], and the majority of the ravens at the Newborough roost also utilize the typical juvenile raven foraging strategy [6]. In addition, we assume that the probability that a carcass is defended by a breeding pair of adult ravens is zero. This assumption follows from the observation that there are no breeding pairs within about 5 km of the main Newborough roost, probably as a result of excessive foraging competition from the high densities of roosting birds [6].

Following this rationale, we assume that juvenile ravens at a communal roost behave so as to maximize:
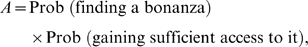
(1)over a single round of the game (a search period, a feeding period and two roosts [14]). Moreover, each bird has an equal probability (λ) of finding a food patch in the time available for searching between roosts; if birds search for food independently of one another, the probability that at least one such bird finds food is an increasing function (S) of the number of birds searching (*k*). Alternatively, if the birds search together in a group then the probability that the group finds a food patch is also an increasing function (G) of the number of birds in the group (*k*). However, the rate of increase with *k* will be lower for G than S (i.e. ∂G/∂*k*<∂S/∂*k*). Specifically:
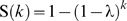
(2)and,
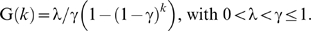
(3)Then:
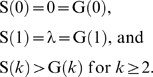
This notation allows for the relative magnitude of the benefits derived from sharing search effort (information sharing) to be specified by λ/γ; the smaller this ratio is, the larger S(*k*) – G(*k*) will be, and hence the better it is to search independently and share carcass encounter information rather than search together in a group.

Without territorial adults to defend carcasses, all birds could potentially gain free access to a located food bonanza. However, we assume that dominant roost members will attempt to exclude other roost mates from the patch [1], [15], with the individual with the most experience at a carcass (e.g. the finder and recruiter) being dominant to all others [4], [6], [9], [17]. Thus, the probability that a subordinate, non-guarding bird will get sufficient access to the food to maintain a positive energy budget (guards always achieve their desired budgets with certainty) is an increasing function (D) of the number of non-guarding birds present (*i*). Specifically:
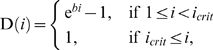
(4)where
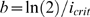
(5)and *i_crit_* is the number of non-guarding birds required to swamp the dominant's ability to control access to the food patch. See Dall [14] for detailed justification of these assumptions.

### The foraging game

We assume that the typical ancestral juvenile raven roost consisted of *n*+1 birds, which are unlikely to have encountered each other in the past or are unlikely to encounter each other again, and individuals will have had widely varying histories of foraging success. Therefore, each bird has an equal chance of being the closest to starvation, and hence being the first bird after the first dawn of a roost's existence to have to leave the roost to forage. Upon the departure of this ‘starter’ bird, the remaining *n* birds choose one of two actions: depart and search for food individually (play S), or follow the starter and search as a group (play F). Furthermore, at the end of the search period, or when a carcass has been located, all birds (including the starter) can choose one of two actions: return to the roost and attempt to recruit others (or be recruited) to a carcass at the subsequent dawn (play R), or roost as near as possible to the located carcass (or where they ended up at the end of the day) and do not actively recruit (or be recruited by) any other birds (play D). The choice of actions affects the dominance status of individuals at a carcass in the following ways: (a) groups of successful searchers (≥2) will always recruit individuals to their carcass thereby ensuring they have ‘the most experience’ at the carcass and gain the ‘dominance advantage’; (b) if multiple birds locate the same carcass, those individuals that remain closest to the carcass (play D) will be dominant; (c) only one individual is ever dominant at a carcass, and when multiple individuals ‘have the most experience’ based on (a) and (b), dominant status is assigned randomly (i.e. other than relative experience and a ‘sheep effect’, the factors that determine dominance vary at random with respect to the actions chosen by the birds). We assume that the ravens will always play (unconditionally) one of S or F at the roost, and R or D at the patch/end of the search period. See Dall [14] for detailed justification of these assumptions. Thus, there are four potential foraging strategies that compete over evolutionary time in the juvenile common raven system, defined in Table 1.

**Table 1:** the strategy set.

Strategy	Definition
SR	Leave the roost and search independently, return to roost and recruit (or be recruited) at the end of the search period
SD	Leave the roost and search independently, do not return to roost and do not recruit (or be recruited) at the end of the search period. Roost where finish search period (i.e. near any located carcass).
FR	Leave the roost and follow the ‘starter’ bird and search as a group, return to roost and recruit at the end of the search period
FD	Leave the roost and follow ‘starter’ as a group, do not return to original roost and do not recruit at the end of the search period. Roost in group where finish search period.

Given the above formulation, following Dall [14] and by analogy with and Mesterton-Gibbons and Milner-Gulland [18], we model the strategic interaction as a symmetric (*n*+1)-player game, reduced effectively to a two-player game by assuming that a focal (mutant) individual interacts with the *n* remaining birds, which are all assumed to be identical. Player 1 is the mutant individual, and Player 2 is the rest of the group; symmetry implies that the choice of focal individual is arbitrary. The matrix of rewards per round of play to a mutant individual using a given row strategy in a population using a given column strategy can therefore be determined from (1)–(5), and is shown in Table 1 and 2 in Tables S1. We denote this matrix by **A**, so that *a_IJ_* is the reward (in terms of (1)) to a mutant individual playing *I* against *n* individuals playing strategy *J*. Since our formulation is equivalent to that in Dall's [14] ‘Scenario 2’, ignoring the possibility of action ‘W’ (wait) at the roost and *p* (the probability that a carcass is defended by a territorial pair of adults) = 0, we refer readers to that paper for details of how the expressions in Tables 1 and 2 in Tables S1 are derived.

### Conditions for strategic stability

A strong (symmetric) Nash-equilibrium strategy (or strong evolutionarily stable strategy- ESS [19]) of such a game is a population strategy that is also uniquely the focal individual's best reply to the other *n* players of it. Thus, a population strategy is stable if its diagonal element in Table 1 in Tables S1 is the largest in its column. In other words, strategy *J* is stable if *a_JJ_* exceeds *a_IJ_* for all *I*≠*J*.

## Results

From our formulation, as in Dall's [14] ‘Scenario 2’, two strategies emerge as strongly evolutionarily stable: SR and FD (search independently and recruit; and follow from the roost and do not return, recruit or be recruited: Table 1). We proceed by describing how FD, a ‘gang’ foraging equilibrium – similar to the foraging behavior observed in the two Newborough sub-roosts – can be selected for over SR, which is equivalent to the recruitment-based foraging typical of juvenile common ravens in the main roost at Newborough and other roosts.

### ‘Gang’ foraging can invade recruitment-based foraging close to a large roost…

If adult raven pairs abandon their territories in the vicinity of large, stable roosts of juveniles (i.e. *p*→0 in the Dall [14] notation), as appears to be the case at the Newborough roost [6], inspection of the reward matrix in Table 1 in Tables S1 reveals that the typical (ancestral) juvenile raven foraging behavior (SR) can be vulnerable to invasion by gang-based foraging. Specifically, only FR (follow from a roost and return to recruit or be recruited) can invade SR since *a*_11_>*a*_41_ and *a*_11_>*a*_21_ throughout Tables 1 and 2 in Tables S1. The following trade off determines whether this occurs. On the one hand, since an FR mutant always follows the starter bird and searches in a pair, its average search efficiency at a roost is reduced relative to the typical SR player in the population: G(2)+{1−G(2)}S(*n*−1)<S(*n*+1). However, such a mutant will also stand more of a chance than a typical SR player of being dominant if it locates a carcass. This is because searching in a pair increases the chances of it locating a carcass (G(2)>λ), and, if it does so, it will also tend to be more likely to recruit others and be dominant (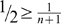). However, without the potential to be dominant at a carcass – when *n*≥*i*_crit_ – this advantage will never confer any fitness benefit in terms of (1) and therefore FR mutants will never invade populations of SR individuals. Below then, we specify the factors that will tip the balance of this trade-off in favor of gang-foraging mutants when there are benefits to being dominant at carcasses (*n*<*i*_crit_: Table 1 in Tables S1).

**Figure 1:**
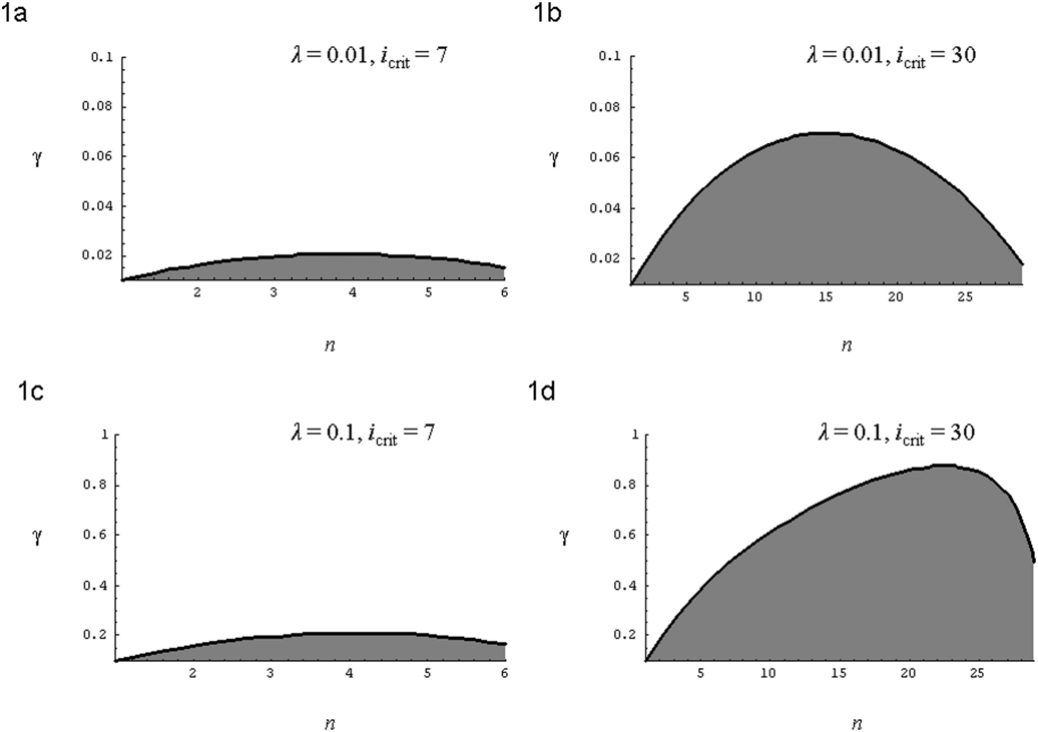
Conditions under which the typical searching-individually-and-recruiting strategy of juvenile common ravens (SR) can be invaded by searching-in-gangs (FR: shaded regions) when there are no non-roost members defending carcasses: (a) λ = 0.01, *i*_crit_ = 7; (b) λ = 0.01, *i*_crit_ = 30; (c) λ = 0.1, *i*_crit_ = 7; (d) λ = 0.1, *i*_crit_ = 30. The thick line plots values of γ for which *a*_31_ = *a*_11_ (Table 1a in Tables S1). Note that the y-axis scales from λ to 0.1 in (a) and (b), while in (c) and (d) it scales from λ to 1, and, the larger γ is relative to λ, the less efficient searching in a group is relative to searching independently and pooling the effort. All figures were drawn with Mathematica [27].

Generally speaking, the less that searching in a group reduces the efficiency of sharing individual search effort, the better-off FR mutants are in SR populations. In other words, FR will invade at smaller γ>λ, as illustrated in Figure 1. Thus, when searching in a pair is almost as good as two birds searching individually and sharing findings (λ/γ→1); the benefits from being likely to be dominant at a carcass can outweigh the efficiency costs of searching in a pair. In addition, FR mutants are most likely to invade at intermediate roost sizes relative to the critical number of birds required to nullify the ‘dominance advantage’ (*i*_crit_), and when carcasses are relatively common (e.g. at intermediate *n* relative to *i*_crit_ and large λ in Figure 1). This is because, under both such conditions, the efficiency costs of searching in a pair are again diminished, and the dominance advantage is still significant. On the one hand, as roost size grows, the impact of losing just two independent searchers and having them search together on the likelihood of at least one roost member finding a carcass becomes less significant. Moreover, in addition to having their search efficiency costs reduced, successful FR mutant searchers are much more likely to recruit and be dominant at larger roosts (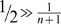 as *n*→∞). However, as roost sizes approach *i*_crit_ the dominance advantage also becomes less and less significant as it becomes increasingly difficult to restrict roost mates' access to a carcass. Indeed, this dynamic also means that increasing the number of birds required to swamp dominance at the carcass will increase the opportunities for FR mutants to invade. This is because increasing *i*_crit_ allows larger roost sizes to be tolerated before the dominance advantage is eroded and, when *n*≪*i*_crit_, subordinates have relatively little chance of gaining sufficient access to a located carcass, thereby increasing the premium associated with being dominant. Alternatively, when carcasses are common, being relatively inefficient at searching matters less, which also undermines the efficiency cost of searching as a pair. Overall, then, gang-based foraging from fixed sub-roosts can be favored over the more typical individual recruitment based foraging when group searching is relatively efficient and/or carcasses are relatively common, and such roosts are sizeable and dominant ‘finders’ are effective at preventing the other, subordinate roost mates from accessing carcasses reliably (*a*_31_>*a*_11_ in Table 1 in Tables S1 under such conditions).

### …and can be stable at intermediate roost sizes

**Figure 2:**
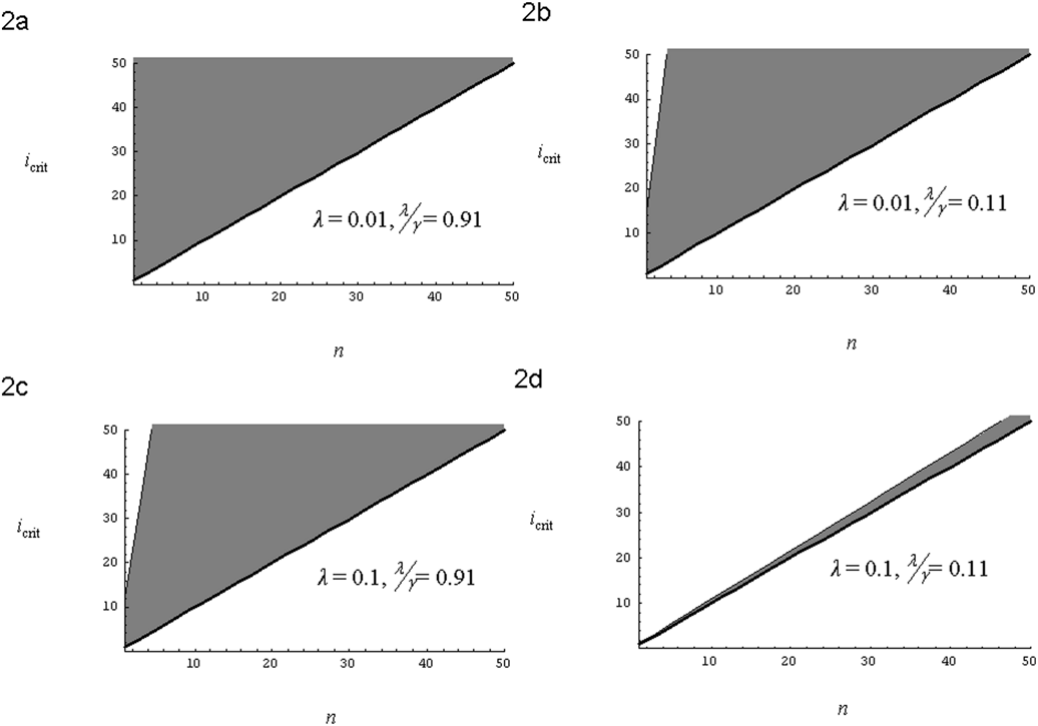
Conditions under which searching-in-gangs is likely to be observed when there are no non-roost members defending carcasses: (a) λ = 0.01, γ = 0.011, (b) λ = 0.01, γ = 0.09, (c) λ = 0.1, γ = 0.11, (d) λ = 0.1, γ = 0.9. The darker shading denotes where FD is a strong ESS, while the unshaded regions are where SR is likely to invade and spread to fixation (resist reinvasion). The thin and thick lines represent values of *i*_crit_ for which *a*_24_ = *a*_44_ and *a*_34_ = *a*_44_ (*n* = *i*_crit_) respectively (Table 1 in Tables S1). Above the thick line dominants are relatively effective at excluding subordinates and there is therefore a ‘dominance advantage’ (*n*<*i*_crit_), while below there is no such advantage (*n*>*i*_crit_).

Once FR mutants invade populations of SR players (as discussed above), FD (foraging in gangs and not returning to a particular roost to recruit or be recruited; Table 1) will invade the emergent populations of fixed-roost FR gang foragers (*a*_43_≥*a*_33_ throughout Table 1 in Tables S1). This is because FD mutants will always be dominant at a carcass by roosting nearby in splinter roosts after the gang has located it, thereby gaining more experience at it than the average FR player. The conditions under which gang foraging (with or without a fixed roost) is likely to resist reinvasion by the more typical, recruitment-based foraging are illustrated in Figure 2. A crucial factor determining the persistence of gang-based foraging is the relationship between splinter-roosting gang-size (*n*) and the critical number of birds required to overcome the carcass defense of dominants (*i*_crit_). Since the latter parameter is unknown for the Newborough ravens (see Discussion), we proceed by discussing separately FD stability when gangs are smaller (Table 1 in Tables S1) and larger than (or equal to) *i*_crit_ (Table 2 in Tables S1).

If gangs, which roost together at splinter roosts, are smaller than the critical number of birds required to overcome the carcass defense of dominants (*n*<*i*_crit_), populations playing FD are only vulnerable to invasion by mutants that search individually and do not return, recruit or be recruited (SD; only *a*_24_>*a*_44_ under some conditions in Table 1 in Tables S1). However, this will only ever be the case if gangs are very small compared to *i*_crit_, or carcasses are common and searching in a group is relatively inefficient compared to searching alone. This is illustrated in Figure 2, in the unshaded regions above the thick line, when *n*≪*i*_crit_, or λ is large and λ/γ is small. Under such conditions, the benefit of having free access to carcasses outweighs the costs of having no one to search for food with. This is because SD players are always alone, and in small groups subordinate group members have little chance of gaining access to carcasses that are defended effectively. Therefore solitary foraging can invade gang-based foraging. However, in the long run, if this happens, typical SR foraging is likely to reestablish itself since it can invade SD foraging by drift and will resist reinvasion by any other strategy (*a*_12_ = *a*_22_ and *a*_11_>*a_J_*_1_, where *J* = 2, 3, 4, for *n*≪*i*_crit_ in Table 1 in Tables S1). Overall then, when dominants can defend carcasses effectively, gang-based foraging from independent roosts is likely to persist (be evolutionarily stable) unless gangs are very small, or carcasses are common and searching in a group is inefficient (Figure 2), which is unlikely to be the case close to the main Newborough roost [6].

On the other hand, when carcass defense by dominants is relatively ineffective, and gang sizes typically exceed the number of birds required to overcome carcass defense (*n*≥*i*_crit_), FR can spread through populations of FD foragers by drift (and FD can drift back) since there is no disadvantage (or advantage) to FR mutants missing out on the opportunity to be dominant by roosting furthest from the carcass, on average. However, any emergent FR groups can then be invaded by SR mutants, since such individuals enjoy higher chances of locating carcasses than the average FR players as a result of adding their individual search effort to the gang's: G(*n*)+{1−G(*n*)}λ>G(*n*+1). If this happens, SR will spread to fixation (and resist reinvasion): *a*_43_ = *a*_33_ = *a*_34_ = *a*_44_, *a*_13_>*a*_33_ and *a*_11_>*a*_31_ in Table 1b in Tables S1 when *n*≥*i*_crit_. Thus, based on our formulation, we expect in the long run not to observe gang-based foraging when dominants are relatively ineffective at excluding subordinates from carcasses since the information center benefits to the typical juvenile common raven foraging behavior [14] prevail under these conditions.

## Discussion

Although our formulation reveals that the typical juvenile raven foraging strategy of searching individually and recruiting from a communal roost can be vulnerable to invasion by gang-based foraging under a range of conditions (Figure 1), only a subset of these conditions is compatible with the long-term persistence of this foraging strategy (Figure 2). Given the conditions observed around the Newborough roost then, this work suggests that the sub-roosts, from which gangs of ravens apparently forage for carrion locally around the main raven roost [6], exist for the following reasons. In general, without territorial adults attempting to defend carcasses, gang-based foraging from independent roosts is likely appear and persist when searching in groups is not particularly inefficient compared to individuals searching independently and sharing their effort by recruiting from a central roost. This will be the case if the area searched is not particularly large (e.g. can be searched by a group in a day) and/or it is in open habitat, within which carcasses are visible from a distance – in open habitats black vultures typically forage in groups from communal roosts [20]–[23]. Indeed, the habitat around the Newborough roost consists of open woodland, coastal beaches, rocky shores, sand dunes and agricultural pasture. Moreover, sub roost birds typically only forage in the area immediately around the roost, which can easily be searched by a gang over a day (no pairs defend territories within ∼5 km of the roost [6]).

Our analysis also reveals that another key issue is the productivity of the habitat. The agricultural landscape in North Wales means that a greater number of carcasses are available than would have been ancestrally. Indeed, Ratcliffe [2] and Wright et al. [6] suggest that raven roosts are so large and stable in Europe, compared to the small, transient roosts in the forests of New England, because food is relatively plentiful and nearby. On the one hand, this bounty may have contributed to likelihood that the ancestral search-independently-and-recruit foraging strategy was invaded by gang-foraging by undermining the value of sharing information acquired independently (compare (a), (b) with (c), (d) in Figure 1). However, plentiful food also weakens the stability of gang-foraging against solitary foragers that become more and more likely to find food without ever having to risk having their access to it restricted in the absence of adults defending food patches (Figure 2: (a), (b) versus (c), (d)). This trade off may determine the minimum size of the areas over which stable foraging gangs can operate – gang territories must not be so small that solitary foragers can search them as effectively as a group can. How big this is depends on the productivity of the habitat and how easy it is to search. This suggests that future empirical work should be directed to quantifying the likelihood of finding food per unit of raven foraging time, both when solitary and in groups.

Furthermore, our model predicts that evolutionarily stable foraging gangs, which roost separately from other birds in splinter roosts, are likely to be moderately sized relative to the critical number of birds required to overcome the efforts of dominant finders/recruiters to exclude other roost mates (the strength of dominance). If they are too small (i.e. dominance effects are strong), gang-foraging mutants may be able to spread only to lose out when they become common to solitary foragers that avoid the risk of ever being subordinate at a carcass. On the other hand, if gang-roosts are too large (i.e. dominance is weak), they will not be strongly evolutionarily stable and the ancestral foraging mode will reestablish itself since returning to a main roost will not be selected against. The two sub roosts close to the main Newborough roost consist of about 30 birds each, which is a moderate size given the size of the main roost – 500 to 1500 birds [6]. Moreover, although estimates are currently unavailable for the maximum number of birds whose access to food an at-a-carcass dominant juvenile can even partially limit (*i*_crit_); it is unlikely to exceed 30. Indeed, observations both in New England and North Wales indicate that it takes about 7 birds to overcome the resource defense of the breeding pair whose territory the carcass is on [4], [6]. However, since carcass defense by dominant juvenile birds may function to increase their overall social status and attractiveness in a mate choice context [1], [6], [15], as well as to secure access to food, it is likely to take more than 7 birds to discourage committed attempts at carcass defense. Nevertheless, our analysis suggests that this parameter is a key influence on juvenile common raven foraging behavior and should therefore also be a focus for future empirical work.

It is clear that the model presented here is relatively simplistic and could be further developed in a number of ways. The original Dall [14] model that this work is based on was built around observations of extreme vagrancy by the juvenile ravens populating communal roosts [1], [7], resulting in very ephemeral roost composition and little opportunity for repeated interactions between players. This, in turn, made unconditional strategies a realistic simplification since maintaining behavior that is best on average at roosts makes sense adaptively under such conditions. However, the Newborough roost is relatively stable in its composition, being populated by birds that are either year-round residents or over-winter visitors [6]. This suggests that allowing for repeated interactions between players and considering strategies that are conditional on experience, for instance, would be of value in future theoretical work. Repeated social interactions will clearly have an influence on the evolution of strategies for the gathering and sharing of foraging information, as well as the collective defense of food bonanzas. This seems especially important here because of the clear dominance relationships that exist between ravens foraging cooperatively together, not to mention the possibilities for social reputations and relationships leading to breeding opportunities [1], [6]. Indeed, it is likely that allowing for dominance to be a function of repeated interactions between individuals will strengthen gang foraging from regular roosts (FR) relative to gangs with relatively mobile roosts (FD). This is because ‘experience at a carcass’ will lose its preeminence in determining dominance and therefore FD mutants will find it difficult to invade FR gangs that have invaded ancestral populations of individual-searching-and-recruiting foragers. This may help to explain why foraging gangs have been observed to operate exclusively from two regular sub roosts in Newborough [6], however further theoretical work is required to confirm this intuition. Indeed, this would require a very different approach to that adopted here, in which individual experience is modelled explicitly (e.g. using state dependent dynamic programming [24] with ‘experience’ or status as an organismal state). In addition, introducing a spatial element into a model of this type would also seem very appropriate, given the geographical structuring of large European raven roosts like that in Newborough [6]. Spatial factors also seem important if we are to explore the differences between the extensive natural habitats of North America versus the mostly agricultural habitats of Europe. A spatially explicit model would therefore provide a useful ecological context for the information center hypothesis, and more generally for the gathering and social transmission of information concerning food locations (e.g. [25]).

In conclusion, by refocusing a general model of juvenile common raven foraging behavior [14]) on the conditions under which newly documented yet theoretically anticipated gang-based foraging has been observed at a raven roost in North Wales [6], we hope to have shed further light on the complex social foraging behavior of this ‘feathered primate’. On the one hand, we show formally how the trade off between search efficiency and social opportunity may account for the existence of the alternative social foraging tactics observed in this species. In the process, our analysis also highlights key issues that should motivate future empirical effort. Nevertheless, our analysis may also have implications for other social foragers on food resources that occur in ephemeral hyper-productive patches (e.g. fruiting trees, marine pelagic prey etc.). Such resources bring ICH benefits into play [14], [25] and social trade offs can predominate over energetic ones [14]. Under such conditions, our analysis suggests that stable foraging groups should evolve when (a) groups can exclude individual foragers from food patches and individuals within groups gain social status over group mates via ‘finder effects’ [26], (b) food patches are moderately difficult to find (too easy or too difficult and it pays individuals to forage alone) and (c) groups are moderately sized so that individuals can sometimes but not always dominate other group members. Overall then, by exploring the search efficiency vs social status trade off in the simplest possible game – single, randomized interactions between unconditional strategies – we have identified likely key ecological influences on juvenile common raven foraging behavior at winter roosts. Nevertheless, as in any successful modeling exercise, it is where our formulation falls short that highlights the gaps in our understanding of the system, and how to plug them. There is still plenty to be done to elucidate this fascinating system!

## Supporting Information

Tables S1 (0.06 MB PDF)
