# Supplementary material for: Rich Pickings Near Large Communal Roosts Favor ‘Gang’ Foraging by Juvenile Common Ravens, Corvus corax
Source: PLoS One. 2009 Feb 25;4(2):e4530. doi: 10.1371/journal.pone.0004530 (PMC2646834; doi:10.1371/journal.pone.0004530)
Supplement: Tables S1 — (0.06 MB PDF) [file pone.0004530.s001.pdf]

Dall & Wright Supplementary Information: Payoff Tables

Table 1: payoffs to mutants playing row strategies against populations of *n* column strategists with a 'dominance advantage'; i.e. for $n < i = i_{crit}$

	SR	SD	FR	FD
SR	$\frac{\left(1+\left(-1+2^{\frac{n}{i}}\right) n\right)\left(1-(1-\lambda)^{1+n}\right)}{1+n}$	λ	$\frac{1}{(1+n)\gamma}\left(\lambda\left(\frac{1-(1-\gamma)^{1+n}}{1+n}+\frac{1}{1+n}\left(\left(-1+2^{\frac{n}{i}}\right) n\left(1+n+n^2-(1-\gamma)^n\left(1+n+n^2-\gamma\right)\right)\right)+n\left(\gamma+\left(-1+(1-\gamma)^n\right)\lambda\right)\right)\right)$	$\frac{\left(n+\frac{\left(-1+2^{\frac{n}{i}}\right)\left(1-(1-\gamma)^{1+n}\right)}{\gamma}\right)\lambda}{1+n}$
SD	λ	λ	$\frac{\left(n+\frac{1-(1-\gamma)^{1+n}}{\gamma}\right)\lambda}{1+n}$	$\frac{\left(n+\frac{\left(1+\left(-1+2^{\frac{n}{i}}\right) n\right)\left(1-(1-\gamma)^{1+n}\right)}{\gamma}\right)\lambda}{1+n}$
FR	$\frac{1}{1+n}\left(\frac{1-(1-\lambda)^{1+n}}{1+n}-\frac{1}{2} n(-2+\gamma)\lambda+\left(-1+2^{\frac{n}{i}}\right) n\left(1+\frac{1-(1-\lambda)^{1+n}}{1+n}-(1-\lambda)^{-1+n}+\lambda-\frac{\gamma\lambda}{2}\right)\right)$	$\frac{1}{1+n}\left(\left(1-\left(-1+2^{\frac{n}{i}}\right) n(-2+\gamma)\right)\lambda\right)$	$\frac{\left(1+\left(-1+2^{\frac{n}{i}}\right) n\right)\left(1-(1-\gamma)^{1+n}\right)\lambda}{(1+n)\gamma}$	$\frac{\left(-1+2^{\frac{n}{i}}\right)\left(1-(1-\gamma)^{1+n}\right)\lambda}{\gamma}$
FD	$\frac{1}{(1+n)^2}\left(1-(1-\lambda)^n+(1-\lambda)^n\left(\left(-1+2^{\frac{n}{i}}\right) n(-1+\lambda)+\lambda\right)+n\left(-1+2^{\frac{n}{i}}-(1+n)(-2+\gamma)\lambda\right)\right)$	$\frac{\left(2-2^{\frac{n}{i}} n(-2+\gamma)\right)\lambda}{2(1+n)}$	$\frac{\left(1-(1-\gamma)^{1+n}\right)\lambda}{\gamma}$	$\frac{\left(1+\left(-1+2^{\frac{n}{i}}\right) n\right)\left(1-(1-\gamma)^{1+n}\right)\lambda}{(1+n)\gamma}$

Dall & Wright Supplementary Information: Payoff Tables

Table 2: payoffs to mutants playing row strategies against populations of n column strategists without a ‘dominance advantage’; i.e. for $n \geq i = i_{crit}$

	SR	SD	FR	FD
SR	$1-(1-\lambda)^{1+n}$	λ	$(\lambda(1+n^2+n\gamma-n\lambda+(1-\gamma)^n(-1+\gamma+n(-n+\lambda))))/((1+n)\gamma)$	$\dfrac{\left(n+\frac{1-(1-\gamma)^{1+n}}{\gamma}\right)\lambda}{1+n}$
SD	λ	λ	$\dfrac{\left(n+\frac{1-(1-\gamma)^{1+n}}{\gamma}\right)\lambda}{1+n}$	$\dfrac{n\lambda}{1+n}+\dfrac{\lambda-(1-\gamma)^{1+n}\lambda}{\gamma}$
FR	$((n+(-1+\lambda)^2)(1-\lambda)^n-(-1+\lambda)(-1+n(-1+(-2+\gamma)\lambda)))/((1+n)(-1+\lambda))$	$\dfrac{(1-n(-2+\gamma))\lambda}{1+n}$	$\dfrac{(1-(1-\gamma)^{1+n})\lambda}{\gamma}$	$\dfrac{(1-(1-\gamma)^{1+n})\lambda}{\gamma}$
FD	$-\dfrac{-1+(1-\lambda)^{1+n}+n(-2+\gamma)\lambda}{1+n}$	$\dfrac{(1-n(-2+\gamma))\lambda}{1+n}$	$\dfrac{(1-(1-\gamma)^{1+n})\lambda}{\gamma}$	$\dfrac{(1-(1-\gamma)^{1+n})\lambda}{\gamma}$
